# Periodontal disease and inflammatory blood cytokines in patients with stable coronary artery disease

**DOI:** 10.1590/1678-775720160082

**Published:** 2016

**Authors:** Cassio KAMPITS, Marlon M. MONTENEGRO, Ingrid W. J. RIBEIRO, Mariana V. FURTADO, Carisi A. POLANCZYK, Cassiano K. RÖSING, Alex. N HAAS

**Affiliations:** 1 - Universidade Federal do Rio Grande do Sul, Faculdade de Odontologia, Departamento de Periodontia, Porto Alegre, RS, Brasil.; 2 - Universidade Federal do Rio Grande do Sul, Faculdade de Medicina, Divisão de Cardiologia, Porto Alegre, RS, Brasil.

**Keywords:** Cytokines, Cardiovascular diseases, Periodontal diseases, Atherosclerosis, Interleukins

## Abstract

Periodontal disease has been associated with elevations of blood cytokines involved in atherosclerosis in systemically healthy individuals, but little is known about this association in stable cardiovascular patients. The aim of this study was to assess the association between periodontal disease (exposure) and blood cytokine levels (outcomes) in a target population of patients with stable coronary artery disease (CAD).

**Material and Methods:**

This cross-sectional study included 91 patients with stable CAD who had been under optimized cardiovascular care. Blood levels of IL-1β, IL-6, IL-8, IL-10, IFN-γ, and TNF-α were measured by Luminex technology. A full-mouth periodontal examination was conducted to record probing depth (PD) and clinical attachment (CA) loss. Multiple linear regression models, adjusting for gender, body mass index, oral hypoglycemic drugs, smoking, and occurre:nce of acute myocardial infarction were applied.

**Results:**

CAD patients that experienced major events had higher concentrations of IFN-γ (median: 5.05 pg/mL vs. 3.01 pg/mL; p=0.01), IL-10 (median: 2.33 pg/mL vs. 1.01 pg/mL; p=0.03), and TNF-α (median: 9.17 pg/mL vs. 7.47 pg/mL; p=0.02). Higher numbers of teeth with at least 6 mm of CA loss (R2=0.07) and PD (R2=0.06) were significantly associated with higher IFN-γ log concentrations. Mean CA loss (R2=0.05) and PD (R2=0.06) were significantly related to IL-10 concentrations. Elevated concentrations of TNF-α were associated with higher mean CA loss (R2=0.07).

**Conclusion:**

Periodontal disease is associated with increased systemic inflammation in stable cardiovascular patients. These findings provide additional evidence supporting the idea that periodontal disease can be a prognostic factor in cardiovascular patients.

## INTRODUCTION

Inflammation plays an important role in the initiation and development of atherosclerosis^19^. Atherosclerotic lesions are comprised of various cell types that produce pro- and anti-inflammatory cytokines, which participate in the plaque formation cascade in vessel walls^12^. Specifically, interleukin (IL)-6, tumor necrosis factor (TNF)-a, and interferon (IFN)-γ are found in elevated concentrations in blood from cardiovascular patients^22,29^, and the IL-10 concentration is increased in unstable compared with stable atheromas^18^. Moreover, IL-6 and TNF-a have direct effects on the production and release of C-reactive protein (CRP)^20^ and, consequently, have been used as predictors of future cardiovascular events in cardiac patients^11^.

Periodontal diseases are caused by bacteria attached to teeth in organized biofilms and is characterized by bleeding, loss of tooth surrounding tissues, and tooth mobility, being the major cause of halitosis and tooth loss. Periodontal diseases may be expressed using various clinical parameters and definitions by which they are associated with risk factors and systemic conditions^26^. Probing depth and clinical attachment loss are the most frequent periodontal parameters used to define periodontal health/disease status, and they may be expressed as individual averages or by the number of teeth affected *per* individual.

One of the possible mechanism linking periodontal diseases to cardiovascular diseases (CVD) is the low-grade inflammatory effect resulting from the periodontal etiopathogenic process^14,22^. Studies have demonstrated that periodontitis, which is the destructive form of periodontal disease, increases systemic levels of some of the aforementioned CVD-related cytokines^3,15^. Most evidence supporting this claim has been provided by studies conducted in otherwise systemically healthy individuals, suggesting that periodontal disease may be a risk factor for CVD. On the other hand, there is little information about the systemic effects of periodontal disease on patients with stable CVD, with few studies evaluating the association between periodontal disease and a small number of systemic inflammatory biomarkers in cardiovascular patients^2,8,10,17,28^.

To the best of the authors’ knowledge, no study has evaluated the association between periodontal parameters and blood levels of a broad range of cytokines, assessed by Multiplex technology, in cardiovascular patients. Such studies may indicate a prognostic effect of periodontal inflammation and destruction in the occurrence of future cardiovascular events on these patients. Therefore, the aim of this study was to assess the associations between periodontal disease and blood cytokine levels in patients with stable CAD who were under standard cardiovascular care.

## MATERIAL AND METHODS

This cross-sectional observational study was conducted with stable CAD patients who had been receiving cardiovascular care for at least six months in a tertiary care cardiovascular clinic at a university hospital in Brazil. To be included in the study, all patients had to present with CAD, defined as the occurrence of at least one of the following events six months before entering the study: documented history of myocardial infarction (MI), stable angina, or ischemia in noninvasive tests; surgical or percutaneous myocardial revascularization and lesion size greater than 50% in at least one major coronary artery, as assessed by angiography; and presence of angina and positive results of noninvasive testing of ischemia. Additional inclusion criteria included the presence of at least four teeth, no periodontal treatment, and no use of antibiotics or anti-inflammatory drugs for six months prior to the study.

Overall, 239 consecutive patients were evaluated from April to December 2011. Among them, 78 (32.6%) individuals had less than four teeth, 30 (12.6%) refused participation, and 17 (7.1%) lived outside the city and were not available for clinical examination. Moreover, 16 (6.7%) did not attend the clinical examination appointment. Clinical examinations were performed in 98 (41.0%) individuals; however, seven did not undergo laboratory blood analysis. The final sample of the present investigation comprised 91 individuals.

The study protocol was approved by the institutional review boards of the University Hospital and the Federal University of Rio Grande do Sul. All participants read and signed an informed consent form before entering the study.

### Cardiovascular care

All patients received cardiovascular tertiary care including medication and counseling. Regarding drug prescription, the protocol of the cardiovascular care in this clinic includes statins for the majority of patients. When appropriate, oral hypoglycemic drugs, insulin, acetylsalicylic acid, and antihypertensives were also prescribed. Counseling mainly includes daily exercise, smoking cessation, and dietary therapy. Most patients in this sample were using statins (92.3%), acetylsalicylic acid (90.1%), and antihypertensive drugs (93.4%). Only 13.1% and 9.9% were using insulin and antidepressives, respectively.

### Data collection

We applied a structured questionnaire to collect data about age, gender, and smoking exposure. We obtained medical history, medication use, weight, and height from the hospital records of the patients.

Clinical periodontal parameters were assessed with a manual periodontal probe (Williams probe, Newmar, São Paulo, SP, Brazil) by two calibrated examiners (weighted kappa values ranging from 0.80 and 0.90) who were unaware of the cardiovascular systemic inflammatory condition of the patients. Visible plaque (VP), gingival recession (GR), probing depth (PD), and bleeding on probing (BOP) were recorded at six sites *per* tooth in all present teeth, excluding third molars. Clinical attachment (CA) loss was obtained as the sum of GR and PD.

### Blood samples

A trained nurse collected 20 mL of blood from the antecubital fossa of each individual. Fasting blood samples were obtained between 7:00 am and 12:00 pm to control for possible diurnal variations. Half of the blood (10 mL) was stored in EDTA-containing Eppendorf tubes and was immediately centrifuged for analysis. Plasma from the remaining 10 mL was stored at -80°C for quantification of cytokines. Samples were stored for two years on average.

### Cardiovascular status

The cardiovascular status of patients was assessed by identifying known blood markers of risk for cardiovascular events. High-sensitivity CRP, glucose, glycated hemoglobin, triglycerides (TG), total cholesterol (TC), and high-density lipoprotein cholesterol (HDL-C) were measured as previously described^8^. Low-density lipoprotein cholesterol (LDL-C) was calculated by the Friedwald formula.

Participants were further divided into those having medium- and long-term past history of major cardiovascular events using a combination of information about the occurrence of cardiovascular events and time elapsed since the event. Patients that have experienced acute myocardial infarction or myocardial revascularization (angioplasty or surgery) in the last nine years were included in the high status group, whereas if the events occurred before 10 years or more, the individuals were classified in the low status group.

### Systemic cytokines

Plasma levels of IL-1β, IL-6, IL-8, IL-10, IFN-γ, and TNF-α were measured in uniplicate by Luminex methodology in combination with the 3.1 Xponent software package (Luminex Corp., Austin, TX, USA ). The MILLIPLEX MAP Human Cytokine/Chemokine Magnetic Bead Panel-07 kit (HCYTOMAG-60k, EMD Millipore, Saint Charles, USA) was used following the manufacturer’s instructions. Results were expressed as standard curve units in pg/mL. All samples were analyzed at the same time under standardized experimental conditions.

In summary, before starting the immunoassay, samples were completely thawed, mixed by vortexing, and centrifuged at 1000g for 10 minutes to remove particulates. All kit reagents were prewarmed at room temperature. A 200-μL aliquot of wash buffer was added to each of the 96 wells in the plate, which was then sealed, mixed on a plate shaker for 10 minutes at room temperature, inverted, and tapped several times onto absorbent towels to decant and remove the residual wash buffer. The standard curve and control wells received 25 μL of standard and/or control solutions. The remaining wells received 25 μL of the samples. Assay buffer alone was used as the background (0 pg/μL). Finally, 25 μL of assay buffer were added to all wells, for 50 μL *per* well.

Each antibody-bead vial was sonicated for 30 seconds and vortexed for 1 minute. Then, 60 μL from each antibody-bead vial were added to the mixing bottle to bring the final volume to 3 mL with bead diluent. Next, 25 μL of the bead dilution were added to each well. The plate was sealed and incubated overnight (18 hours) at 4°C with agitation on a plate shaker. Well contents were gently removed, and the plate was washed twice with wash buffer. A 25-μL aliquot of detection antibodies was added to each well and incubated for 1 hour at room temperature with agitation on a plate shaker. Without further washing, 25 μL of streptavidin-phycoerythrin were added to each well, followed by incubation for another 30 minutes with agitation on a plate shaker. Washing with wash buffer was repeated twice. Finally, 150 μL of sheath fluid were added to all wells. The beads were re-suspended on a plate shaker for 5 minutes, and the plate was run on Luminex. The median fluorescent intensity was analyzed by using a five-parameter logistic curve-fitting method for calculating cytokine concentrations in the samples.

### Statistical analysis

The outcomes of the present study were blood levels of inflammatory cytokines. Models were separately fitted for each of the following cytokines: IL-1β, IL-6, IL-8, IL-10, IFN-γ, and TNF-α. Cardiovascular blood markers (CRP, TG, lipids, glucose, glycated hemoglobin) were expressed as means and standard deviations (SD) to describe the cardiovascular profile of the sample.

Numbers of teeth with PD and CA loss of 6 mm or greater were calculated. Individuals were classified as having severe periodontitis in the presence of two or more interproximal sites with CA loss of 6 mm or greater and at least one interproximal site with PD of 5 mm or greater in nonadjacent teeth^7^.

Multiple linear regression models were used to study the association between periodontal status and systemic levels of cytokines. In these models, periodontal status was defined by four criteria: mean CA loss, mean PD, and numbers of teeth with CA loss of at least 6 mm and PD of at least 6 mm. Cytokine concentrations were log-transformed (base 10). Predictive models were fitted by including periodontal status as the main independent variable and adjusting them for gender, body mass index (BMI), use of oral hypoglycemic drugs, lifetime smoking exposure (in packyears), and time elapsed since the occurrence of acute myocardial infarction. Residuals were used to assess the fitness of the models. Regression coefficients, standard errors (SE), and adjusted R^2^ values were determined. It has been suggested that the levels of triglycerides may modify the production of cytokines^5^. Thus, an exploratory analysis was performed by stratified comparisons according to the TG control status, with 150 mg/dL as the cut-off.

Secondary analysis was performed using diagnostic tests to estimate the magnitude severe periodontitis that may predict higher levels of systemic cytokines. Cytokine levels were dichotomized by the median value. Sensitivity, specificity, and positive and negative predictive values (PPV and NPV, respectively) were calculated for severe periodontitis and each cytokine.

Statistical analyses were performed with a statistical package (STATA SE for Macintosh version 13, StataCorp, College Station, USA). The individual was the unit of analysis. The alpha level was set at 5%.

## RESULTS

The mean age of the sample was 62.9 (SD: 9.9) years and the average lifetime smoking exposure equaled 21.8 (SD: 31.9) packyears. Table 1 describes the characteristics of the sample. Considering the observed mean values, cardiovascular patients from the present study had elevated levels of CRP and TG, whereas TC, HDL, and LDL were mostly under control. Low levels of IL-1β and IL-6 were observed. Overall, 68 (76.4%) individuals had severe periodontitis.

**Table 1:** Demographic and behavioral characteristics of the study sample (n=91)

Characteristic	n (%)
Male gender (n/%)	54 (59.3)
Smoking exposure (n/%)	
Non-smoker	35 (38.4)
Former smoker	46 (50.6)
Smoker	10 (11.0)
BMI (Kg/m2)	27.84±4.56
**Periodontal status**	Mean ± SD
Visible plaque (%)	67.9±20.3
PD (mm)	3.0±0.7
CA loss (mm)	4.8±1.6
BOP (%)	74.7±24.2
Tooth loss (number of teeth)	12.9±7.0
**Cardiovascular variables**	Mean ± SD
CRP (mg/L)	4.50±5.25
Triglycerides (mg/dL)	179±132
Total cholesterol (mg/dL)	167±30
LDL-C (mg/dL)	89±28
HDL-C (mg/dL)	42±12
Glucose (mg/dL)	122±46
Glycated hemoglobin (%)	6.98±2.07
**Cytokines**	Mean ± SD
IFN-γ (pg/mL)	5.23±9.55
IL-10 (pg/mL)	4.79±12.97
IL-1β (pg/mL)	1.26±2.79
IL-6 (pg/mL)	2.81±2.61
IL-8 (pg/mL)	6.65±5.64
TNF-α (pg/mL)	10.09±5.80

A total of 52 (57.1%) patients experienced acute myocardial infarction, and more than half of these patients had this major event during the nine years before the study (medium-term past history of events). CAD patients in the medium-term past history, compared with the long-term group, had significantly higher concentrations of IFN-γ, IL-10, and TNF-α (Table 2).

**Table 2:** Concentrations of blood cytokines according to cardiovascular status of the sample (values expressed as median, minimum – maximum)

	Past history of cardiovascular events	
Cytokines	Long-term	Medium-term
IFN-γ (pg/mL)	3.01 (0.76-34.13)	**5.06 (0.90-84.89)**
IL-10 (pg/mL)	1.01 (0.05-20.06)	**2.33 (0.05-88.47)**
IL-1β (pg/mL)	0.66 (0.05-4.7)	0.71 (0.05-26.46)
IL-6 (pg/mL)	1.92 (0.59-15.60)	2.13 (0.38-11.70)
IL-8 (pg/mL)	4.45 (1.45-30.00)	4.97 (1.53-27.82)
TNF-α (pg/mL)	7.47 (4.00-34.02)	**9.17 (2.81-63.18)**

Bold numbers indicate significant differences between groups of cardiovascular status (p<0.05)

There were significant associations between periodontal parameters and IFN-γ, IL-10, and TNF-α (Table 3). High numbers of teeth with CA loss (R^2^=0.07) and PD of at least 6 mm (R^2^=0.06) were significantly associated with higher IFN-γ concentrations. Mean CA loss (R^2^=0.05) and PD (R^2^=0.06) were significantly related to the IL-10 concentration. Elevated TNF-α concentrations were associated with higher mean CA loss (R^2^=0.07). There were no significant associations between periodontal parameters and IL-1β, IL-6, and IL-8.

**Table 3:** Multiple linear regression models of the association between periodontal status and serum cytokines (log transformed), with adjustment for gender, body mass index, use of oral hypoglycemic drugs, smoking exposure, and time elapsed since major cardiovascular event

	Mean CA loss		Mean PD		Teeth with CA loss ≥6 mm		Teeth with PD ≥6 mm	
Cytokine	beta±SE	R2	beta±SE	R2	beta±SE	R2	beta±SE	R2
IFN-γ	0.02±0.02	0.03	0.05±0.04	0.04	**0.02±0.01**	**0.07**	**0.02±0.01**	**0.06**
IL-10	**0.07±0.03**	**0.05**	**0.12±0.06**	**0.06**	0.02±0.01	0.04	0.03±0.02	0.05
IL-1β	0.001±0.03	0.03	0.002±0.06	0.03	-0.01±0.01	0.04	-0.01±0.02	0.03
IL-6	0.02±0.02	0.02	0.03±0.04	0.02	0.003±0.01	0.01	0.01±0.01	0.02
IL-8	0.01±0.02	0.05	-0.03±0.03	0.06	0.01±0.01	0.05	-0.01±0.01	0.06
TNF-α	**0.02±0.01**	**0.07**	0.02±0.02	0.04	-0.001±0.01	0.03	-0.002±0.01	0.03

Bold numbers indicate statistical significance (p<0.05)

Table 4 shows multiple linear regression models of the association between clinical attachment loss and serum cytokines (log transformed) according to triglyceride control. In individuals with poorly controlled TG (≥150 mg/dL), the number of teeth with CA loss of at least 6 mm was significantly associated with IFN-γ, IL-10, and IL-1β concentrations. No significant associations were found for IL-6, IL-8, and TNF-α.

**Table 4:** Multiple linear regression models of the association between clinical attachment loss and serum cytokines (log transformed) according to triglyceride control

	Number of teeth with CA loss ≥6 mm			
	Triglycerides ≥150 mg/dL		Triglycerides <150 mg/dL	
Cytokine	beta±SE	R2	beta± SE	R2
IFN-γ	0.04±0.02	0.19	0.01±0.01	0.08
IL-10	0.03±0.01	0.1	0.01±0.02	0.02
IL-1β	0.06±0.03	0.1	0.01±0.01	0.17
IL-6	0.001±0.01	0.04	0.003±0.01	0.03
IL-8	0.001±0.01	0.18	0.01±0.01	0.06
TNF-α	0.01±0.01	0.24	0.001±0.01	0.04

Diagnostic tests of severe periodontitis as a predictor of cytokine concentrations above the median are shown in Table 5. A positive diagnosis of periodontitis indicated sensitivity values ranging between 77.8 and 84.4, whereas the absence of periodontitis was related to low values of specificity (range: 20.0–31.8). PPVs were close to 50.0, and NPVs varied between 38.1 (IL-1β) and 66.7 (IFN-γ).

**Table 5:** Diagnostic tests of severe periodontitis as a predictor of cytokine concentrations above the median (95% confidence interval in parentheses)

	Sensitivity	Specificity	Positive predictive value	Negative predictive value
IFN-γ	84.4 (70.5-93.5)	31.8 (18.6-47.6)	55.9 (43.3-67.9)	66.7 (43.0-85.4)
IL-10	77.8 (62.9-88.8)	25.0 (13.2-40.3)	51.5 (39.0-63.8)	52.4 (29.8-74.3)
IL-1β	73.5 (58.9-85.1)	20.0 (9.1-35.6)	52.9 (40.4-65.2)	38.1 (18.1-61.6)
IL-6	77.8 (62.9-88.8)	25.0 (13.2-40.3)	51.5 (39.0-63.8)	52.4 (29.8-74.3)
IL-8	82.6 (68.6-92.2)	30.2 (17.2-46.1)	55.9 (43.3-67.9)	61.9 (38.4-81.9)
TNF-α	77.8 (62.9-88.8)	25.0 (13.2-40.3)	51.5 (39.0-63.8)	52.4 (29.8-74.3)

## DISCUSSION

The present study demonstrated that periodontal disease is associated with systemic inflammation in patients with chronic CVD. Specifically, poor periodontal condition, as measured by CA loss and PD, was associated with increased blood levels of IFN-γ, IL-10, and TNF-α. These cytokines were also related to major events in the studied sample. These findings provide additional evidence supporting the notion that periodontal disease may be a prognostic factor in cardiovascular patients.

The role of IL-10 in the cascade of atherosclerosis and cardiovascular events remains controversial, with some studies indicating that high IL-10 concentrations may have a protective effect on MI^9,23^ and other longitudinal observational studies^4,16^ reporting that baseline elevated blood IL-10 levels increase the risk for MI. This controversy seems to have been clarified by a recently published meta-analysis^13^, which demonstrated that high IL-10 serum levels are significantly associated with new cardiovascular events over time in patients with acute coronary syndrome. If IL-10 is truly related to new major cardiovascular events, then periodontal disease might have a detrimental effect on atherosclerosis by increasing the levels of IL-10, as demonstrated in this study. Nevertheless, the possible effect of other confounders not assessed in the present study and which may be related to the association found between periodontal status and IL-10 should not be discarded.

IFN-γ and TNF-α are proatherogenic cytokines^21^. IFN-γ acts on smooth muscle cells by inhibiting collagen production and smooth cell proliferation, thereby altering the plaque composition and stability^30^. TNF-α is involved in the stimulation of IL-6 production^27^. In the present study, clinical parameters of periodontal inflammation and destruction were significantly associated with IFN-γ and TNF-α. These findings suggest that periodontal disease may act in early stages of the cascade of atherogenic events in cardiovascular patients by increasing the levels of IFN-γ and TNF-α.

The IL-6 cytokine is directly involved in the hepatic production of CRP^20^. Periodontal disease was not associated with blood levels of IL-6 in the present sample of cardiovascular patients. However, very low levels of this cytokine were detected in our patients, probably due to the cardiovascular care received by using statins. Such low levels may have hindered possible associations between periodontal status and this cytokine.

The TG concentration was an effect modifier of the association between periodontal tissue breakdown and some cytokines in this study. The systemic inflammatory effect of periodontal attachment loss on IFN-γ, IL-10, and IL-1β was only significant in patients with high TG levels. Previous findings have also suggested some modulatory effect of elevated TG levels on the production of some cytokines when stimulus came from *P. gingivalis* lipopolysaccharides^5^. Taken together, these data may indicate that the effect of periodontal disease on systemic inflammation can differ with different levels of cardiovascular control. Nevertheless, this effect should be explored in the future for better understanding its significance in the management of this patient population.

A diagnosis of severe periodontitis was correlated with above-median concentrations of cytokines by calculating the sensitivity, specificity, PPV, and NPV in an attempt to provide a better estimation of the clinical relevance of the associations found in the linear regression models that used log-transformed data (which cannot be directly interpreted). It was evidenced that performing a periodontal examination for establishing the presence of periodontitis may be recommended because sensitivity values were close to 80%, indicating that the presence of periodontitis will most often be related to above-median cytokine concentrations. On the other hand, when the diagnosis of periodontitis was already defined as present, the prediction probabilities of above-median concentrations of cytokines were low (PPV range: 51.5–55.9). When the diagnosis was defined as the absence of periodontitis, the prediction of low levels of IFN-γ performed better (66.7) compared with other cytokines.

There is extensive evidence suggesting that periodontal disease may be a risk factor for CVD^26^. In contrast, evidence is still emerging to determine if periodontal disease may be a prognostic factor in patients already diagnosed with coronary disease. Risk factors and prognostic factors differ in that the first is related to the establishment of disease, whereas the second relates to the course of an existing disease^1^. We obtained the findings of the present study in chronic CAD patients. Thus, the main focus here was to study periodontal disease in the presence of cardiovascular disease, potentially attributing a perspicuous factor in the disease process. It will be reasonable to assume that such marker (periodontal disease) could play a prognostic role in the history of CVD. Noteworthy, this study is the first to associate periodontal disease parameters with a broad range of blood cytokines, as quantified by Luminex in CAD patients. Previous studies support a possible role of periodontal disease in the course of CVD by finding associations between periodontal parameters and IL-6 and TNF-α^10,17,25^.

Findings of the present study should be analyzed in view of its possible limitations. The results of the stratified analysis should be interpreted with caution because of the loss of statistical power. Another limitation may include the cross-sectional nature, which precludes causal inferences from being drawn from the associations. Luminex analyses were performed in uniplicate because of costs, and this may have lowered the precision of the readings. The storage time of the samples of two years may be a limitation; however, there is evidence that the cytokines quantified in this study remain stable during this period of time^6,31^.

This study applied well-established methodologies, which strengthens its findings. Cytokines were measured by the Multiplex technology that allows fast, reliable, and accurate quantification of biomarkers in the blood. The analyses controlled for possible confounding effects by applying multivariable models. Moreover, periodontal examinations were conducted using a full-mouth protocol of six sites per tooth to avoid assessment bias^24^. A broad range of periodontal parameters was used to define the periodontal status of the sample and to associate parameters with blood levels of cytokines.

## CONCLUSIONS

Periodontal disease is associated with increased systemic inflammation in cardiovascular patients. Greater CA loss and PD were associated with higher blood levels of IFN-γ, IL-10, and TNF-α, even after controlling for important confounders.
